# Intra-Uterine Perforation Presented as a Giant Cystic Abdominal Mass in a Neonate: A Giant Meconium Pseudocyst

**DOI:** 10.7759/cureus.33625

**Published:** 2023-01-10

**Authors:** Chetna Rathi, Kiran Khedkar, Sagar Karotkar, Raju K Shinde, Yashwant Lamture

**Affiliations:** 1 General Surgery, Jawaharlal Nehru Medical College, Datta Meghe Institute of Higher Education and Research, Wardha, IND; 2 General Surgery/Pediatric Surgery, Jawaharlal Nehru Medical College, Datta Meghe Institute of Higher Education and Research, Wardha, IND; 3 Pediatrics, Jawaharlal Nehru Medical College, Datta Meghe Institute of Higher Education and Research, Wardha, IND

**Keywords:** neonates, cystic mass, fetoreduction, meconium peritonitis, meconium pseudocyst

## Abstract

A meconium pseudocyst is formed following meconium peritonitis. At present, antenatal diagnosis and planned management of meconium pseudocyst have reduced the mortality rate significantly. We presented a case of a neonate with abdominal distension and non-passage of meconium who experienced respiratory distress and was taken for exploratory laparotomy at a tertiary care center due to suspected bowel perforation. The neonate was diagnosed with a meconium pseudocyst intraoperatively as maternal ultrasound and ultrasound of the abdomen of the neonate after birth failed to make a definitive diagnosis; even an X-ray abdomen did not reveal pathognomonic egg-shell calcification. An interesting aspect of this case is the mother’s complex obstetric history, which compelled us to conjecture whether it was possible to predict the chances of meconium peritonitis and take steps to prevent it. It must be noted that, despite rigorous research, the researchers could not find reliable literature co-relating the obstetric history of the mother with the formation of a meconium pseudocyst in the neonate.

## Introduction

Meconium pseudocyst formation is the consequence of the in-utero intestinal obstruction or ischemia of the bowel leading to perforation. Leaked meconium is sterile but causes a chemical irritation of the peritoneum. To control the leaked meconium, a pseudocyst is formed. The affected infant may experience non-passage of meconium, abdominal distension, guarding, or rigidity [1-3].

It has been possible to make an antenatal diagnosis of a meconium pseudocyst can through antenatal ultrasound scans of the mother since 1980. This procedure has reduced the mortality rate from 50% to 11%; in addition to diagnosis, it has also assisted in deciding the need for post-natal surgery in 50% of the patients with persistent ascites and signs of obstruction such as dilated bowel loops [4]. A more recent study conducted by Ping et al. [5] in 2017 revealed a similar finding that prenatal ultrasonography successfully diagnosed 83.3% of the patients and improved survival by 94.4%. The scan has high specificity (100%) but low sensitivity (22.2%). The principle of management of meconium cysts is the removal of all debris and the restoration and maintenance of bowel continuity as soon as possible. Although the mortality rate has reduced, no proper study has provided guidelines for the management of meconium pseudocysts.

## Case presentation

A 35-year-old Indian female with a history of gravida 3 Para 1 live 1 abortion 1 came to the emergency department at a rural hospital in Central India following pre-term labor pain and gave birth to a 2 kg female neonate via lower-segment cesarean section. On further detailed inquiry, her history of ovulation induction followed by twin gestation was revealed. Since the woman already had birthed a male child with Duchenne muscular dystrophy, she underwent selective fetoreduction at the gestational age of 14 weeks. The scan at the gestational age of 21 weeks was suggestive of marginal placenta previa grade 1, and she was diagnosed with hypothyroidism at 34 weeks of pregnancy. The mother underwent multiple prenatal ultrasonography, but none were suggestive of fetal bowel obstruction. Prenatal ultrasound was done on admission at the gestation age of 34 weeks and six days and reported polyhydramnios with grade 3 placenta previa with the possibility of placenta accreta and small bowel obstruction.

**Figure 1:**
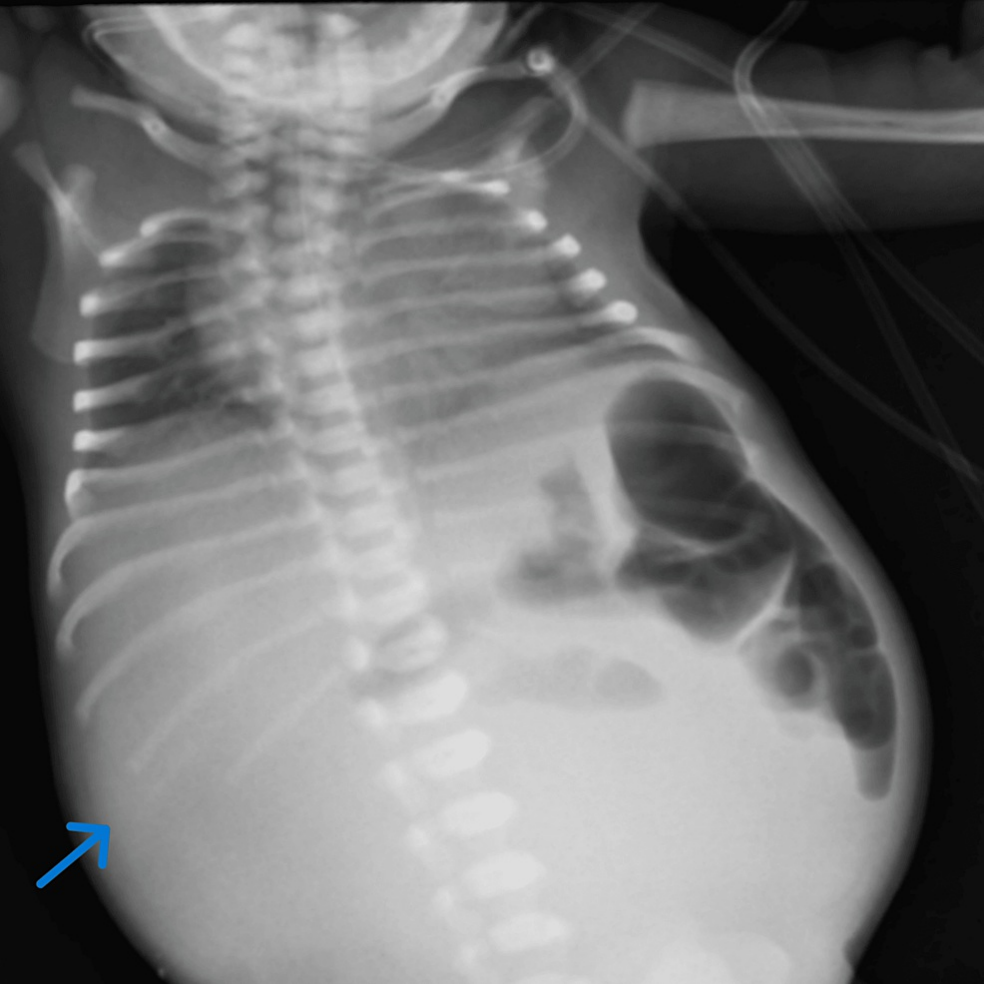
X-ray of a neonate with a meconium pseudocyst resulting from bowel perforation.

Immediately after birth, the female neonate was admitted to the neonatology department of the hospital and was referred to a pediatric surgeon in view of abdominal distension and non-passage of meconium since birth. On examination, the neonate had abdominal distension with erythematous skin over the abdomen, which increased over four hours after birth. The infant feeding tube was also not stained with meconium on per-rectal examination. The nasogastric tube had about 15 ml of bilious aspirate. A roentgenogram (X-ray) of the abdomen in an erect position revealed a large mass lesion on the right side of the abdomen displacing bowel loops on the left side (Figure 1). An ultrasound of the abdomen and pelvis was suggestive of gross free fluid in the abdomen with thick internal echoes. Small bowel dilated with the possibility of perforation. The clinical and radiological findings prompted an immediate exploratory laparotomy.

**Figure 2:**
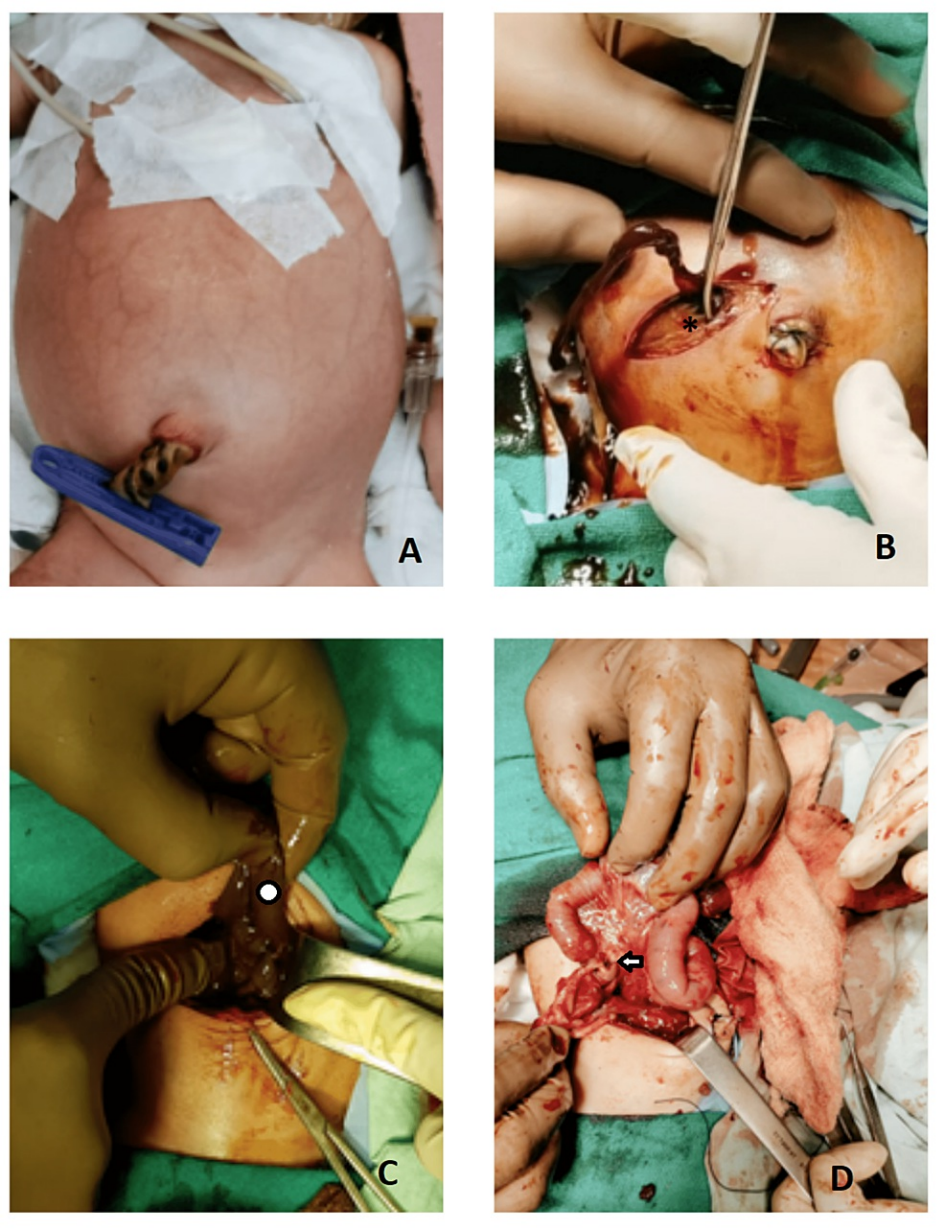
[A] Pre-operative photo of the neonate with abdominal distension; [B] On making an incision, the cyst wall seems to adhere to the abdominal wall (*); [C] Cyst wall (circle) [D] Atretic bowel (arrow).

Exploratory laparotomy was done through a right transverse supraumbilical incision. As soon as the peritoneum was incised, about 200 ml of watery, coffee-ground-colored fluid drained, as the pseudocyst wall was densely adhesive to the abdominal wall and bowel loops. On careful and gentle separation of the pseudocyst wall, the bowel was visible. The bowel was inspected along the length and found to be atretic at the terminal ileum. A short length of the ileum distal to the atretic segment measuring approximately 2 cm along with the cecum and appendix was excised, and a double barrel ileostomy was created (Figure 2). The cyst wall and resected bowel were sent for histopathological examination, which was suggestive of changes of autolysis in the cyst wall; further, resected bowel showed gangrenous necrosis in some parts, and the ileum showed viable mucosa with the normal submucosal gland. 

The neonate did not cry immediately after birth and even after 15 seconds of positive pressure ventilation and was, hence, intubated immediately and shifted to the neonatal intensive care unit. She was started on injectable antibiotics ampicillin and gentamycin along with other supportive care. Post-operatively, she was unable to maintain adequate saturation and blood pressure, and hence, despite all efforts, including inotropic support, the neonate patient succumbed to her illness on postoperative day one. 

 

## Discussion

A meconium pseudocyst is formed following a meconium leak, that is, intrauterine bowel perforation leading to enteric secretions that come in contact with the peritoneal cavity causing a fibrous reaction. It is essentially a fibrous wall formation around the leaked meconium, which is majorly following antenatal bowel obstruction and perforation secondary to meconium ileus, bowel atresia, intussusceptions, Meckel’s diverticulum, Hirschsprung’s disease, idiopathic cause, and sigmoid volvulus [6]

It may present as a palpable abdominal lump, progressive abdominal distension, respiratory distress, or sepsis. According to the meta-analysis conducted by Shinar et al. [7] on fetal meconium peritonitis, prenatal diagnosis was primarily made in the late second or early third trimester, with diagnosis at a mean gestational age of 28.1 ± 2.5 weeks; the strongest indicator of postnatal surgery was meconium pseudocyst. During post-natal diagnosis, occasionally, an abdominal roentgenogram reveals large egg-shell calcification in the case of a long-standing meconium pseudocyst. In Fu et al. [8], the average gestational age for diagnosis of fetal meconium peritonitis was found to be 31.3 ± 4.8 weeks, and the ultrasound revealed intestinal dilation (most common), intraperitoneal calcification, fetal ascites, and intraperitoneal pseudocysts, and polyhydramnios. Some studies claimed that fetal MRI has better diagnostic accuracy between 51% and 73.4% for a fetus with an abdominal cystic lesion and, thus, aids in counseling and planning delivery setting. Fetal MRI is also valuable for predicting postpartum surgical intervention. In Ya et al. [10], it was concluded that surgical intervention should be considered if MRI shows micro-rectocolon and dilated bowel loop along with ascites [10]

Surgery is the standard for the treatment of meconium pseudocysts; surgical procedures consist of drainage procedures and stoma formations, followed by ostomy closure in a second sitting. Notably, a recent retrospective study with 38 patients demonstrated improved survival with primary anastomosis, except for low birth weight babies [11]. The intent of the surgery is to establish intestinal continuity and preserve as much intestinal length as possible. In our case, the meconium pseudocyst was diagnosed on the table as the patient was explored due to suspected perforation. On the discovery of the cyst, a double barrel colostomy was performed through excision of the atretic bowel, appendix, and cecum.

## Conclusions

Based on the case presented, it should be noted that since there was a long complicated maternal history, including fetal reduction, hypothyroidism, and ovulation induction, the etiology for the intestinal perforation causing meconium pseudocyst formation could not be determined. However, one can wonder if the perforation was iatrogenic or spontaneous. Does gestational hypothyroidism in mothers affect bowel growth and development? There is not much evidence supporting and proving all the above claims. Reporting more such cases in detail would help us better understand how to prevent and manage meconium pseudocysts/peritonitis to achieve increased survival among neonates.
